# A Stolen Day in the Country

**Published:** 1907-08-03

**Authors:** 


### Resident Medical Officers' Department.

[Contributions to this Column are invited, and, if accepted, will be paid for.]

### A STOLEN DAY IN THE COUNTRY.

BY A HOSPITAL RESIDENT.

A fine Sunday morning in late May. A week of hard work past, and another week of hard work to come. The sun streams through the open window into my dingy bedroom as I dress after my bath, and a dissolute sparrow twitters in the branches of the solitary plane-tree outside. I begin my breakfast in company with a silent colleague, while one church clock after another chimes out the scandalous hour of ten. Munching my toast, I meditate upon the hard lot of a house surgeon deprived of all the joys of early summer.

"*I've* got to be in all day," remarks my companion, suddenly catching my eye, "but why the blazes *you* don't get out I can't imagine. Make a quick round and put me on duty for you." He pushes towards me an A.B.C. railway guide, and lighting a pipe he lapses once more into silence. I quickly turn up a familiar page and find that there is a train home at 11.15. With a word of grateful assent I hurry off to the wards and impart my project to the sympathetic sisters, in whose company I flit from bed to bed with unaccustomed speed, dressing only two or three grave cases myself and leaving the rest to the willing hands of others. Snatching up pipe, straw hat, and stick, in less than no time I am outside the hospital and hailing a hansom. The cabman, an old patient, whips up his horse, and we gallop through hot and deserted streets to the terminus, pulling up at the entrance with only a minute to spare.

The train glides out over the gleaming river, past factories and squalid dwellings, and soon we are leaving London behind us. Grey gives place to green. I snuff up the warm scent of the country as it blows in through the open window. Flying telegraph poles, momentary visions of cottages and deserted platforms, brief plunges into black tunnels; these were once the dull details of a dull journey; to-day they are the friendly landmarks of a stolen voyage into Arcadia.

At last we draw up at the old station. As I walk up the street I meet the outcoming crowd of worshippers, dressed in Sabbath splendour, and hastening with creaky tread and superb self-satisfaction to the Sunday dinner and the Sunday nap.

Home at last! I open the garden-gate softly, but Jock the terrier has already cocked his ear at an unforgotten step upon the pathway, and, leaving the pursuit of succulent house-flies, he rushes towards me with rapturous welcome. My surprise visit has been well timed, the family have returned from Church and dinner will soon be ready. No prodigal son was ever received with more honour than I after my four months' absence. The rambling old garden has put on its loveliest costume, as if in expectation of my visit. We pace the lawn and each new feature is pointed out and admired. The flower-beds, straggling strips of barren waste at my last visit, are now a blaze of summer tints, from which I reluctantly withdraw my eyes when the gong heralds the dishing up of the fatted calf.

Afterwards an adjournment to the semicircle of hammock chairs beneath the old mulberry-tree whose branches as yet show little more than promise of full foliage. Here, with a flask of wine, a cigarette or two, and an obliging sister playing Mendelssohn in the distant drawing-room, I sit in luxurious indolence, and watch the lilac boughs swaying and the leaves of the lime-trees shivering in the churchyard beyond the garden-wall—and wilderness is paradise enow!

Jock presently demands a walk, so we follow him to the meadows and the rat-haunted streams, sacred to a thousand and one Sunday afternoon rambles. Tea is ready in the garden on our return, and afterwards we play a game of bowls. Afternoon fades into evening. Thrush and blackbird soloists, with full chorus of lesser singers, fill the air with evensong, and one by one retire to rest. The sky takes on an intense shade of blue, and the garden colours fade into primitive effects in neutral tints. Beethoven's sonatas, Chopin's nocturnes, float towards us through the open French windows.

Supper is finished, and it is night. The stolen waters are sinking in the cup, but the last and sweetest drops remain to be drunk. A devious route to the station leads us past a tiny wood. The parish church clock far behind us strikes ten, and then we listen spellbound, as the air throbs with the sustained melody of the nightingale. But a hoarse shriek in the distance breaks the enchantment, and we hurry on to the station to catch the train. A huge basket of flowers—lilac, mauve and white, early lilies of the valley, and late narcissi—fills the carriage with its intoxicating perfume. Soon London is reached, and my stolen day in the country is over.

